# Supplementary figures and images for: Green synthesis of zinc oxide nanoparticles using Catunaregam spinosa (Thunb.) triveng for biologicals applications
Source: PLoS One. 2025 Dec 31;20(12):e0320475. doi: 10.1371/journal.pone.0320475 (PMC12755814; doi:10.1371/journal.pone.0320475)

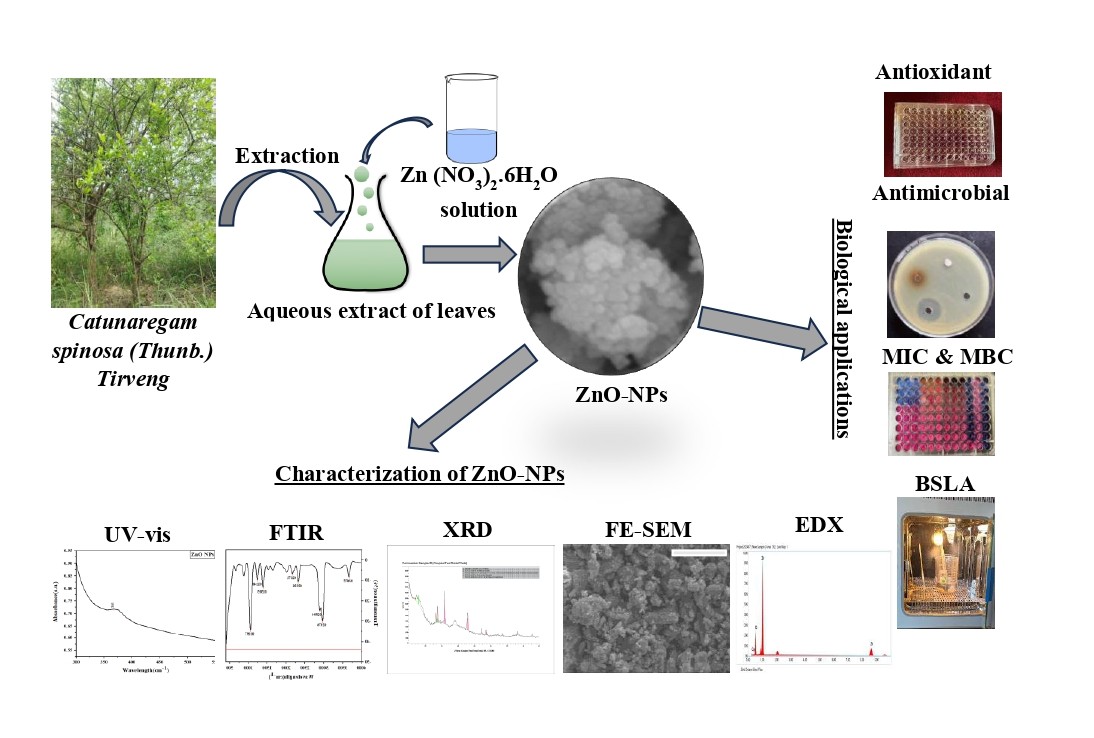

Supplement: S1 Fig — (JPG) [file pone.0320475.s001.jpg]
