# Supplementary material for: Green synthesis of zinc oxide nanoparticles using Catunaregam spinosa (Thunb.) triveng for biologicals applications
Source: PLoS One. 2025 Dec 31;20(12):e0320475. doi: 10.1371/journal.pone.0320475 (PMC12755814; doi:10.1371/journal.pone.0320475)
Supplement: Attachment — Submitted filename: Response to Reviewers.docx [file pone.0320475.s004.docx]

PONE-D-25-09067R1

Green Synthesis of Zinc Oxide Nanoparticles Using Catunaregam spinosa (Thunb.) Triveng for Biologicals Applications

PLOS ONE

Dear Dr. Sharma,

Thank you for submitting your manuscript to PLOS ONE. After careful consideration, we feel that it has merit but does not fully meet PLOS ONE’s publication criteria as it currently stands. Therefore, we invite you to submit a revised version of the manuscript that addresses the points raised during the review process.

Please submit your revised manuscript by Nov 23 2025 11:59PM. If you will need more time than this to complete your revisions, please reply to this message or contact the journal office at [plosone@plos.org](mailto:plosone@plos.org). When you're ready to submit your revision, log on to https://www.editorialmanager.com/pone/ and select the 'Submissions Needing Revision' folder to locate your manuscript file.

Please include the following items when submitting your revised manuscript:

- A rebuttal letter that responds to each point raised by the academic editor and reviewer(s). You should upload this letter as a separate file labeled 'Response to Reviewers'.
- A marked-up copy of your manuscript that highlights changes made to the original version. You should upload this as a separate file labeled 'Revised Manuscript with Track Changes'.
- An unmarked version of your revised paper without tracked changes. You should upload this as a separate file labeled 'Manuscript'.

If you would like to make changes to your financial disclosure, please include your updated statement in your cover letter. Guidelines for resubmitting your figure files are available below the reviewer comments at the end of this letter.

If applicable, we recommend that you deposit your laboratory protocols in protocols.io to enhance the reproducibility of your results. Protocols.io assigns your protocol its own identifier (DOI) so that it can be cited independently in the future. For instructions see: [https://journals.plos.org/plosone/s/submission-guidelines#loc-laboratory-protocols](https://track.editorialmanager.com/CL0/https:%2F%2Fjournals.plos.org%2Fplosone%2Fs%2Fsubmission-guidelines%23loc-laboratory-protocols/1/010f0199c8095fb6-2cc91906-d4f8-4165-ab43-b3886e1c7439-000000/ZHjjWuSaQkufOrqRHZXCnn2uOSD8o-tBSk1JrgNj74E=232). Additionally, PLOS ONE offers an option for publishing peer-reviewed Lab Protocol articles, which describe protocols hosted on protocols.io. Read more information on sharing protocols at [https://plos.org/protocols?utm_medium=editorial-email&utm_source=authorletters&utm_campaign=protocols](https://track.editorialmanager.com/CL0/https:%2F%2Fplos.org%2Fprotocols%3Futm_medium=editorial-email%26utm_source=authorletters%26utm_campaign=protocols/1/010f0199c8095fb6-2cc91906-d4f8-4165-ab43-b3886e1c7439-000000/79vI8XSz8pIu2Q7kGcshvANj8o99LsAk8W-S5bq2814=232).

We look forward to receiving your revised manuscript.

Kind regards,

Muthugounder Subramanian Shivakumar, Ph.D.
Academic Editor
PLOS ONE

Journal Requirements:

If the reviewer comments include a recommendation to cite specific previously published works, please review and evaluate these publications to determine whether they are relevant and should be cited. There is no requirement to cite these works unless the editor has indicated otherwise. 

Please review your reference list to ensure that it is complete and correct. If you have cited papers that have been retracted, please include the rationale for doing so in the manuscript text, or remove these references and replace them with relevant current references. Any changes to the reference list should be mentioned in the rebuttal letter that accompanies your revised manuscript. If you need to cite a retracted article, indicate the article’s retracted status in the References list and also include a citation and full reference for the retraction notice.

Additional Editor Comments (if provided):

Reviewer 2

Authors Sharma et al. revised the manuscript titled Green Synthesis of Zinc Oxide Nanoparticles Using Catunaregam spinosa (Thunb.) Triveng for Biological Applications in response to the reviewers’ comments. The revised version has improved the flow of information and the overall quality of the content, though it still requires some language refinements before acceptance.

Ans: The language has been thoroughly checked in the revised manuscript.

Reviewer 3

The objective of the authors to prepare ZnO NPs using Catunaregam spinosa is fine, but the whole research write-up appears misleading:
Authors must explain why need to choose specific plant part for synthesis. Are there any medicinal properties? If have, is the same compound responsible for reducing Zn ions? How the synchronize for so called biological applications? Whether synthesized material active to absorb UV, authors tested bactericidal properties.

Ans: *Catunaregam spinosa* leaves were selected for the synthesis of nanoparticles due to their abundance of bioactive secondary metabolites, such as flavonoids and phenolic compounds, which serve as organic stabilizing and reducing agents. In addition to being readily available and regenerative, leaves are a sensible and sustainable option for green synthesis. These leaves have been shown to have antibacterial, hepatoprotective, anti-inflammatory, and antioxidant qualities, suggesting the presence of physiologically active substances that can interact with metal ions. The synthesis is directly linked to the biological applications of the nanoparticles because the same bioactive compounds, mainly flavonoids and phenols, are responsible for the reduction of Zn^2+^ ions into nanoparticles and also contribute to the observed antibacterial and antioxidant properties, as per the literature review. Both the photochemical and biological functions of the synthesized nanoparticles are confirmed by their bactericidal activity against both Gram-positive and Gram-negative bacteria, as well as their characteristic UV-Vis absorption peaks in the 300–600 nm range.

1. Can you rethink on title? What indicates biological applications? Why said green synthesis whether your title written as Catunaregam spinosa extract used synthesis? I mean your title must be revised in terms of scientific accuracy.

Ans: The title has been improvised in the manuscript. In our study, "biological applications" refers to testing the produced zinc oxide nanoparticles for toxicity, antibacterial, and antioxidant properties, indicating their potential use in environmental and biomedical domains. We have highlighted key biological activity in the abstract, findings, and discussion sections to make this more evident. At the same time, the phrase "green synthesis" has been kept to highlight that the nanoparticles were made using a sustainable and environmentally benign method, utilizing *Catunaregam spinosa* extract as a biological reducing and stabilizing agent without the use of any dangerous chemicals.

2. Rewrite the abstract, introduction, future prospects, and conclusion in the appropriate direction with addressing the problem specific problem.

Ans: The abstract, introduction, prospects, and conclusion have been revised in the appropriate direction to address the problem-specific in the revised manuscript.

3. Discuss the novelty of your work and similar studies for similar kinds of work here

Ans: Even though nanoparticles have been created utilizing a variety of plant extracts, very little research has been done on nanoparticles synthesis using *Catunaregam spinosa*, specifically for ZnO. This study demonstrates the plant's potential for sustainable nanoparticle production and biological applications by highlighting its rich phenolic and flavonoid content for eco-friendly synthesis. It stands out for its optimized synthesis, comprehensive characterization, and assessment of antibacterial and antioxidant, and toxic properties.

4. Several typos and grammatical errors were found throughout the manuscript; revise it appropriately.

Ans: typos and grammatical errors have been revised appropriately in the revised manuscript.

5. What would be the significance of your studies? What is the strongest report/point?

Ans: The significance of this study lies in presenting an environmentally responsible and sustainable process for creating zinc oxide nanoparticles utilizing leaf extract from *Catunaregam spinosa*, a plant source abundant in phenolic and flavonoid chemicals that serve as organic stabilizing and reducing agents. Significant antibacterial, antioxidant, and toxic properties were demonstrated by the produced nanoparticles, which were well described and suggested prospective uses in environmental remediation, biomedicine, and pharmaceuticals. The use of *Catunaregam spinosa* extract, which has received little attention for the synthesis of green nanoparticles, in conjunction with a methodical assessment of their biological activities, is the most innovative and potent feature of this work. It successfully connects plant bioactive compounds to the formation and functionality of nanoparticles and offers a sustainable and biologically relevant platform for future applications of nanobiotechnology.

Reviewers' comments:

Reviewer's Responses to Questions

**Comments to the Author**

1. If the authors have adequately addressed your comments raised in a previous round of review and you feel that this manuscript is now acceptable for publication, you may indicate that here to bypass the “Comments to the Author” section, enter your conflict of interest statement in the “Confidential to Editor” section, and submit your "Accept" recommendation.

Reviewer #2: All comments have been addressed

Reviewer #3: (No Response)

2. Is the manuscript technically sound, and do the data support the conclusions?

The manuscript must describe a technically sound piece of scientific research with data that supports the conclusions. Experiments must have been conducted rigorously, with appropriate controls, replication, and sample sizes. The conclusions must be drawn appropriately based on the data presented.

Reviewer #2: Yes

Reviewer #3: Yes

3. Has the statistical analysis been performed appropriately and rigorously?

Reviewer #2: (No Response)

Reviewer #3: Yes

4. Have the authors made all data underlying the findings in their manuscript fully available?

The [PLOS Data policy](http://track.editorialmanager.com/CL0/http:%2F%2Fwww.plosone.org%2Fstatic%2Fpolicies.action%23sharing/1/010f0199c8095fb6-2cc91906-d4f8-4165-ab43-b3886e1c7439-000000/kZOzrsFRQ3-vnn4AAR5JR5V-OHxPEmkJkBTzlkB5jOw=232) requires authors to make all data underlying the findings described in their manuscript fully available without restriction, with rare exception (please refer to the Data Availability Statement in the manuscript PDF file). The data should be provided as part of the manuscript or its supporting information, or deposited to a public repository. For example, in addition to summary statistics, the data points behind means, medians and variance measures should be available. If there are restrictions on publicly sharing data—e.g. participant privacy or use of data from a third party—those must be specified.

Reviewer #2: Yes

Reviewer #3: Yes

5. Is the manuscript presented in an intelligible fashion and written in standard English?

PLOS ONE does not copyedit accepted manuscripts, so the language in submitted articles must be clear, correct, and unambiguous. Any typographical or grammatical errors should be corrected at revision, so please note any specific errors here.

Reviewer #2: No

Reviewer #3: No

6. Review Comments to the Author

Please use the space provided to explain your answers to the questions above. You may also include additional comments for the author, including concerns about dual publication, research ethics, or publication ethics. (Please upload your review as an attachment if it exceeds 20,000 characters)

Reviewer #2: Authors Sharma et al. revised the manuscript titled Green Synthesis of Zinc Oxide Nanoparticles Using Catunaregam spinosa (Thunb.) Triveng for Biological Applications in response to the reviewers’ comments. The revised version has improved the flow of information and the overall quality of the content, though it still requires some language refinements before acceptance.

Reviewer #3: The objective of the authors to prepare ZnO NPs using Catunaregam spinosa is fine, but the whole research write-up appears misleading:
Authors must explain why need to choose specific plant part for synthesis. Are there any medicinal properties? If have, is the same compound responsible for reducing Zn ions? How the synchronize for so called biological applications? Whether synthesized material active to absorb UV, authors tested bactericidal properties.
1. Can you rethink on title? What indicates biological applications? Why said green synthesis whether your title written as Catunaregam spinosa extract used synthesis? I mean your title must be revised in terms of scientific accuracy.

2. Rewrite the abstract, introduction, future prospects, and conclusion in the appropriate direction with addressing the problem specifiec problem.
3. Discuss the novelty of your work and similar studies for similar kinds of work here. 4. see some of pubication for further improvement- https://link.springer.com/article/10.1007/s42452-024-06049-z, https://iopscience.iop.org/article/10.1088/2053-1591/ad6ca9/meta, https://www.taylorfrancis.com/chapters/edit/10.1201/9781003181224-13/biogenic-non-biogenic-waste-synthesis-nanoparticles-applications-abhishek-kumar-bhardwaj-ram-naraian-shanthy-sundaram-rupali-kaur 5• Several typos and grammatical errors were found throughout the manuscript; revise it appropriately. 6• What would be the significance of your studies? What is the strongest report/point? Highlight it and make a genuine story to represent your work. It looks messy and fragmented.

Ans: These comments have been incorporated in the revised manuscript.

7. PLOS authors have the option to publish the peer review history of their article ([what does this mean?](https://track.editorialmanager.com/CL0/https:%2F%2Fjournals.plos.org%2Fplosone%2Fs%2Feditorial-and-peer-review-process%23loc-peer-review-history/1/010f0199c8095fb6-2cc91906-d4f8-4165-ab43-b3886e1c7439-000000/QkjWKpy1DFsJIeFXHuZMQiXI5-u4t-AZBh03hPd4plc=232)). If published, this will include your full peer review and any attached files.

If you choose “no”, your identity will remain anonymous but your review may still be made public.

**Do you want your identity to be public for this peer review?** For information about this choice, including consent withdrawal, please see our [Privacy Policy](https://track.editorialmanager.com/CL0/https:%2F%2Fwww.plos.org%2Fprivacy-policy/1/010f0199c8095fb6-2cc91906-d4f8-4165-ab43-b3886e1c7439-000000/FSPcvz8hTs15ltu7S46DMvtExHqpVOrXGzCzNgoNAXM=232).

Reviewer #2: No

Reviewer #3: **Yes:** Abhishek Kumar Bhardwaj, Assistant Professor, Department of Environmental Science, Amity University Madhya Pradesh, Gwalior MP India.

[NOTE: If reviewer comments were submitted as an attachment file, they will be attached to this email and accessible via the submission site. Please log into your account, locate the manuscript record, and check for the action link "View Attachments". If this link does not appear, there are no attachment files.]

While revising your submission, please upload your figure files to the Preflight Analysis and Conversion Engine (PACE) digital diagnostic tool, [https://pacev2.apexcovantage.com/](https://track.editorialmanager.com/CL0/https:%2F%2Fpacev2.apexcovantage.com%2F/1/010f0199c8095fb6-2cc91906-d4f8-4165-ab43-b3886e1c7439-000000/3yFr8iQQhXkJg1FdGjzukOnNUnkCv6jA8GAjrflfw28=232). PACE helps ensure that figures meet PLOS requirements. To use PACE, you must first register as a user. Registration is free. Then, login and navigate to the UPLOAD tab, where you will find detailed instructions on how to use the tool. If you encounter any issues or have any questions when using PACE, please email PLOS at [figures@plos.org](mailto:figures@plos.org). Please note that Supporting Information files do not need this step.
